# Supplementary material for: Arbitrarily routed mode-division multiplexed photonic circuits for dense integration
Source: Nat Commun. 2019 Jul 22;10:3263. doi: 10.1038/s41467-019-11196-8 (PMC6646402; doi:10.1038/s41467-019-11196-8)
Supplement: Supplementary file 1 — Supplementary Information [file 41467_2019_11196_MOESM1_ESM.pdf]

Supplementary Information

Arbitrarily routed mode-division multiplexed photonic circuits for dense integration

Liu *et al.*

Supplementary Note 1 | Three-mode multiplexer

The three-mode (de)MUX was also designed with digital meta-structure via algorithm. The schematic diagram and structural parameters of the designed MUX is described in Supplementary Figure. 1 (a). The simulated in-plane optical field distributions with input wave (TE_0) launched from three different input ports are shown in Supplementary Figure. 1 (b)-(d). The mode profile of each input port is converted into TE_0, TE_1, TE_2 via the 3-mode MUX. Then, we calculate the transmission efficiency of the mode MUX considering the mode overlap in the simulations. As shown in Supplementary Figure 1 (e) - (g), the simulated insertion loss (IL) for each mode is less than 1 dB from 1500 to 1580 nm. The simulated cross talk (CT) s are lower than -20 dB for all modes within the 80 nm wavelength range.

A back-to-back mode MUX is fabricated and the microscope image is shown in Supplementary Figure 2 (a). We fabricate the device by E-beam lithography and dry etching via the in-house facility. The zoom-in SEM image of the nanostructured MUX is shown in Supplementary Figure 2 (b). The transmission spectra of the back-to-back mode MUX are measured and normalized. AS shown in Supplementary Figure 2 (c) – (e), the measured average ILs of TE_0, TE_1, TE_2 modes are all less than 1 dB. The CTs are less than -20 dB in average for all the spatial modes.

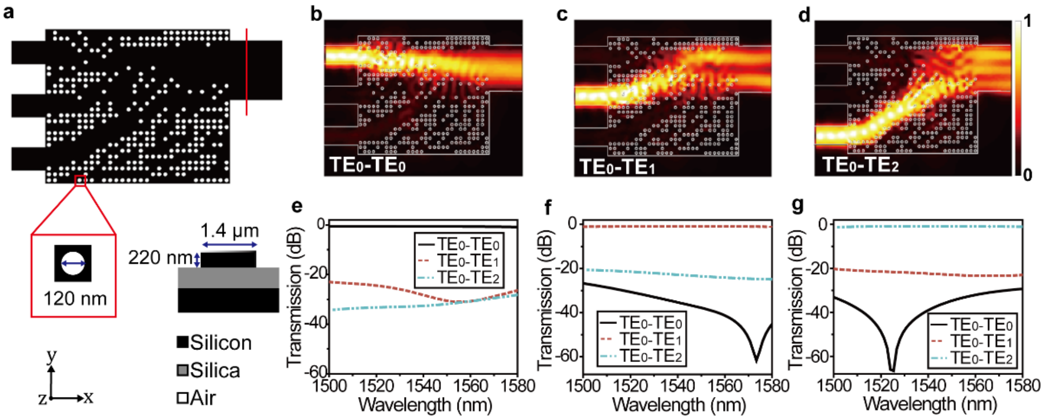

Supplementary Figure 1 | The design and simulation results of the mode MUX. (**a**) Schematic of the designed 3-mode MUX. (**b**) - (**d**) The simulated optical field distribution for the 3-mode MUX when the input wave (TE_0) launch from three different input ports. (**e**) - (**g**) The simulated transmission spectra of the 3-mode MUX.

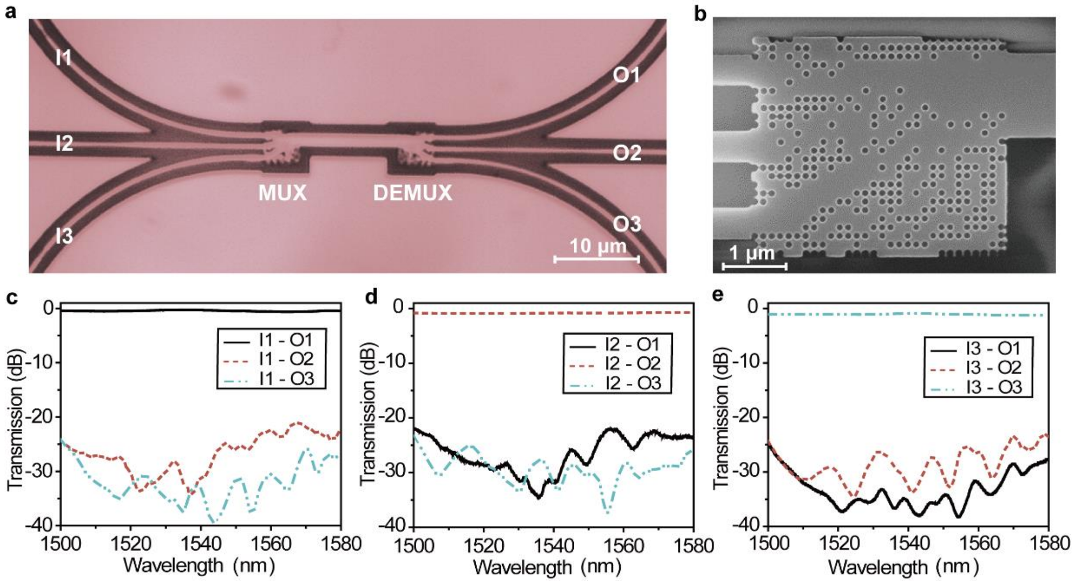

Supplementary Figure 2 | Experimental results of the three-mode MUX. (**a**) The top-view microscope image of the back-to-back (de)MUX circuit. (**b**) The SEM image of the fabricated MUX. (**c**) - (**e**) The measured transmission spectra of the back-to-back (de)MUX circuit for the optical wave launched from I1-I3, respectively.

Supplementary Note 2 | Summary of device performance

Here we summarize the measured transmission performance the different components (3-mode bending, crossing, MUX), MDM1 and MDM2 in Supplementary Table 1. It can be seen that all ILs of the different components are less than 1 dB, which is consistent with the design expectations. The average CTs from 1500 – 1580 nm are lower than -20 dB for most devices. The ILs of MDM1 and MDM2 are consistent with expectations, and the CTs are maintained at a low level as well. (<-20 dB).

Device	ILs (dB)			CTs (dB)					
	TE_0	TE_1	TE_2	TE_0-TE_1	TE_0-TE_1	TE_0-TE_1	TE_0-TE_1	TE_0-TE_1	TE_0-TE_1
3-mode Bending	0.71	0.74	0.95	-20.04	-25.46	-20.01	-25.15	-25.22	-23.53
3-mode Crossing	0.28	0.68	0.92	-23.60	-23.73	-21.10	-22.40	-23.45	-23.27
3-mode MUX	0.68	0.91	0.92	-23.59	-25.64	-21.39	-20.43	-24.98	-20.66
MDM 1	7.36	8.22	8.65	-20.29	-26.79	-21.50	-25.31	-20.04	-20.19
MDM 2	10.67	11.67	13.12	-20.03	-23.62	-24.26	-2137	-20.59	-22.29

Supplementary Table 1 | Summary of the measured ILs and CTs of the devices from 1500 – 1580 nm.

Supplementary Note 3 | Numerical analysis of the fabrication tolerance

The ultra-compact, highly functional and efficient devices are mainly realized by the sophisticatedly engineered distribution of the nanoholes. There are many reasons to cause the variations of the nanoholes in fabrication process, such as improper dose of exposure, development condition, etching rate and so on. Here we numerically analyze the device tolerance to fabrication errors in pixel dimension, position and geometry.

1. The fabrication tolerance of hole dimension

The simulated transmission spectra of the bending under ±20 nm pixel size variations from 1500 nm to 1580 nm for TE_0, TE_1 and TE_2 are shown in Supplementary Figure 3 (a) - (c), respectively. The simulated crosstalk performances of the bending are shown in Supplementary Figure 3 (d) - (i). The results indicate that the ILs for each mode can roughly tolerate -20 to +10 nm pixel size variation. The CTs have lower tolerance than ILs, but the CTs can still be lower than -15 dB for all the cases. Supplementary Figure 4 shows the simulated ILs and CTs of the waveguide crossing under ±20 nm pixel size variations. It can be seen that the ILs of the crossing have no significant change within ±20 nm variations for TE_0 and TE_1, but the transmission performance is very sensitive to pixel size variation for TE_2 mode. With ±20 nm pixel size variations, the CTs can be lower than -20 dB for all the cases. Supplementary Figure 5 shows the simulated ILs and CTs of the (de)MUX under ±20 nm pixel size variations. The ILs of the (de)MUX has very low tolerance since the mode-convert efficiency is very sensitive to the index profile of the multimode region. The CTs of the (de)MUX device can tolerate the hole dimension variation from -10 nm to +20 nm.

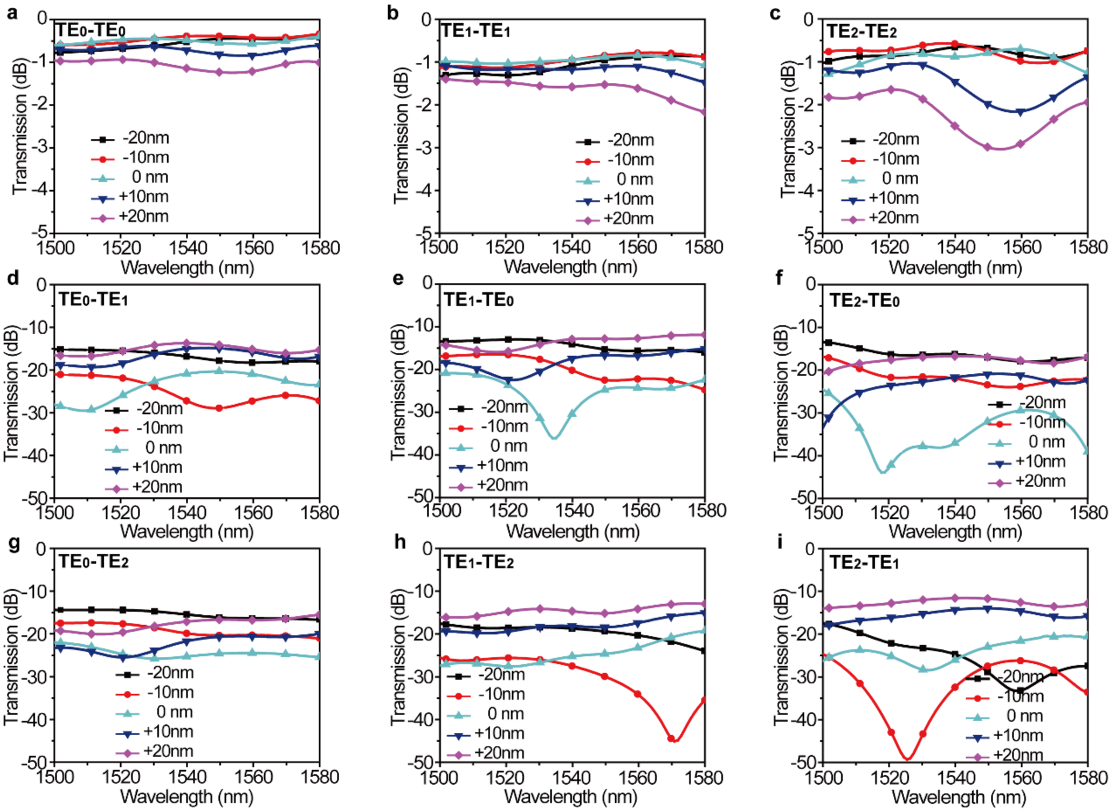

Supplementary Figure 3 | The simulated transmission spectra of the bending under ±20 nm pixel size variations. (**a**) - (**c**) The simulated ILs for (**a**) TE_0 -TE_0 (**b**) TE_1 –TE_1 (**c**) TE_2 –TE_2. (**d**) - (**i**) The simulated CTs for three different modes.

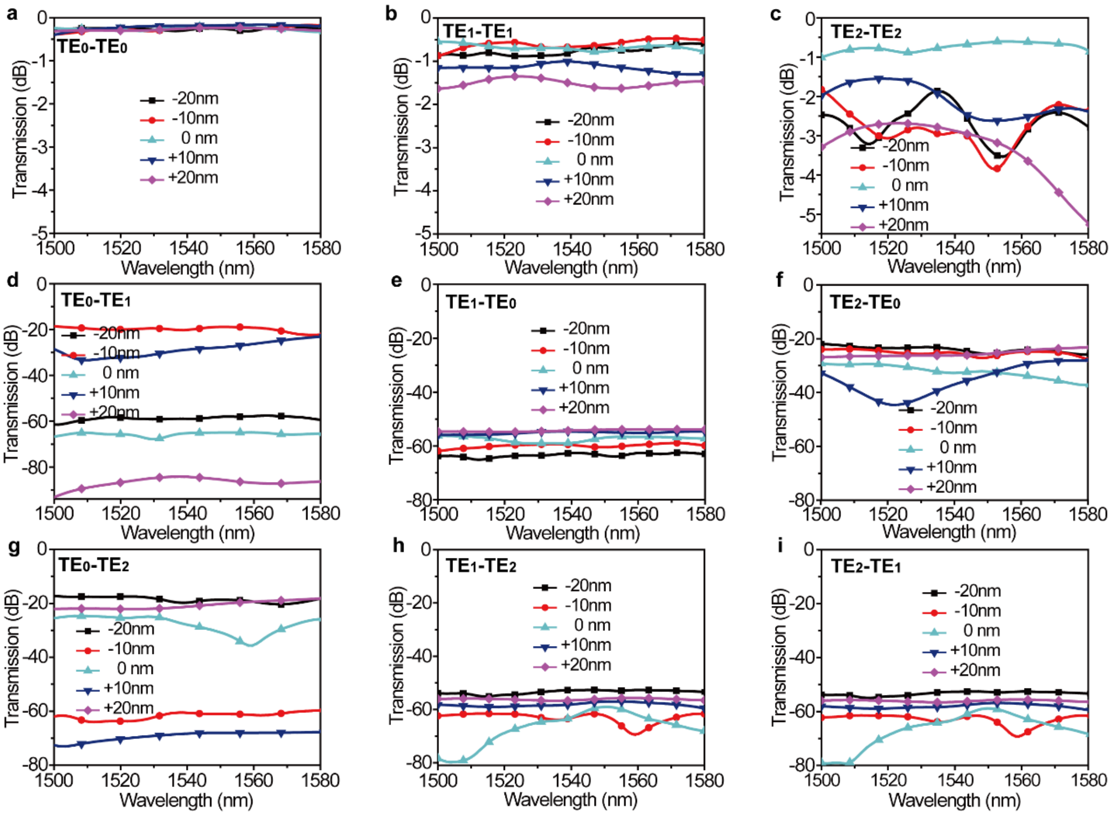

Supplementary Figure 4 | The simulated transmission spectra of the 3-mode crossing under ±20 nm pixel size variations. (**a**) - (**c**) The simulated ILs for (**a**) TE_0 -TE_0 (**b**) TE_1 –TE_1 (**c**) TE_2 –TE_2. (**d**) - (**i**) The simulated CTs for three different modes.

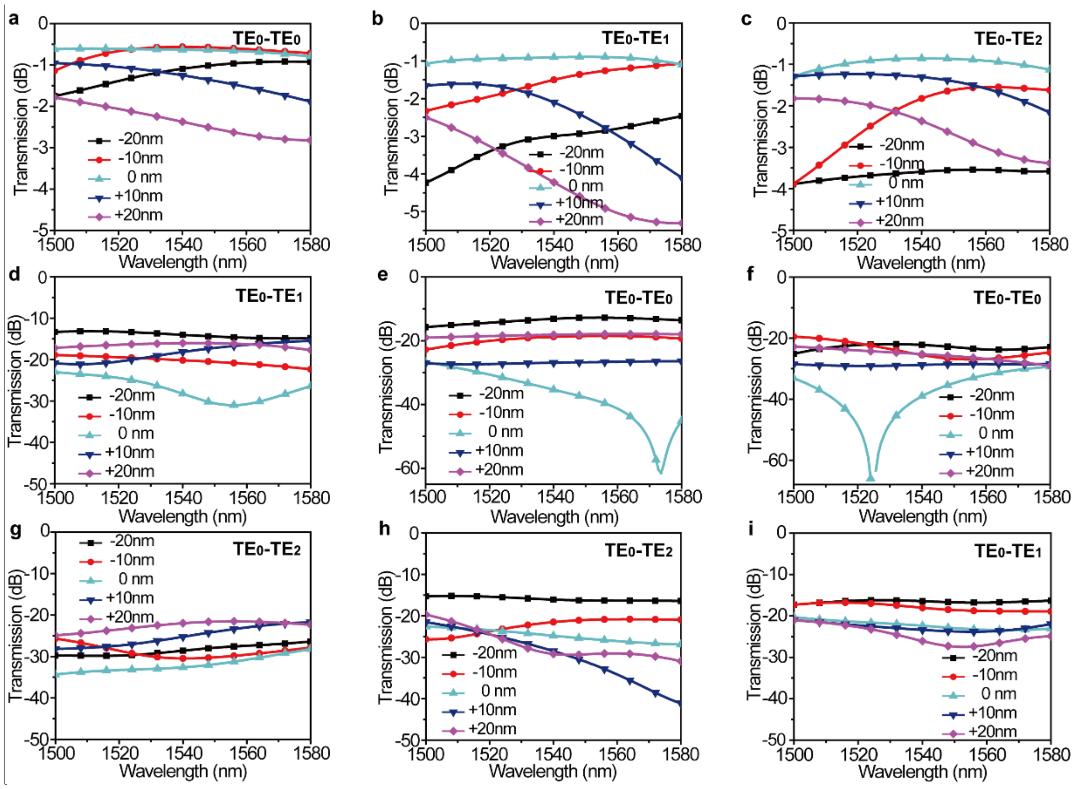

Supplementary Figure 5 | The simulated transmission spectra of the mode (de)multiplexer under ±20 nm pixel size variations. (**a**) - (**c**) The simulated ILs for (**a**) TE_0 -TE_0 (**b**) TE_0 –TE_1 (**c**) TE_0 –TE_2. (**d**) - (**i**) The simulated mode-convert CTs for three different modes.

2. The fabrication tolerance of hole position error.

The hole position error mainly results from the positioning accuracy of the electron gun in our experiment. According to the specification of the E-beam writer, the positioning error is within ±7 nm. As illustrated in Supplementary Figure 6, the center position offset of the nanohole can be in an arbitrary direction. Based on the optimized device pattern, a randomly generated position error is implemented to each nanohole, which will rearrange the overall pattern as shown in Supplementary Figure 6 (b). Then, we select four randomly generated nanohole distributions (defined as Pattern 1, 2, 3, 4), and simulate the device performance. For the waveguide bending, the simulated ILs for different nanohole distributions are shown in Supplementary Figure 7 (a), (b), (c) for TE_0, TE_1 and TE_2 mode, respectively. The CT performances are also simulated and shown in Supplementary Figure 7 (d)-(i). We found that the device can well tolerate the position error of the nanoholes. We have done similar analysis for the waveguide crossing and (de)MUX, as shown in Supplementary Figure 8 and 9, respectively. The results show that the crossing and MUX devices are tolerant to the nanohole position error of ±7 nm as well.

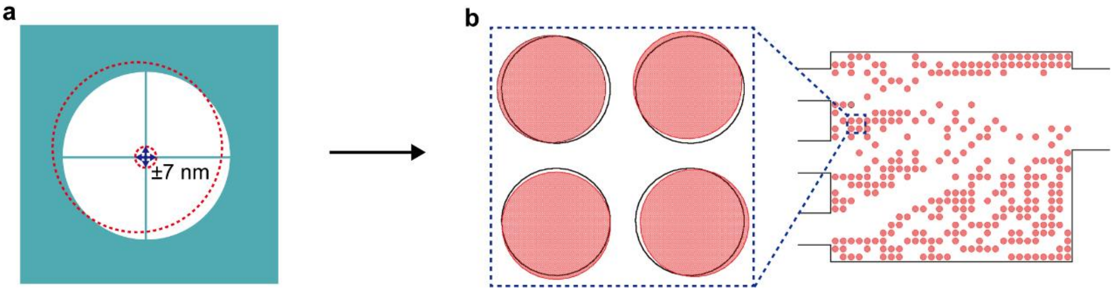

Supplementary Figure 6 | The schematic illustration of hole randomly generated nanohole position error. (a) The illustration of the nanohole position error within ±7 nm in a random direction. (**b**) The schematic diagram of the device with randomly generated position errors.

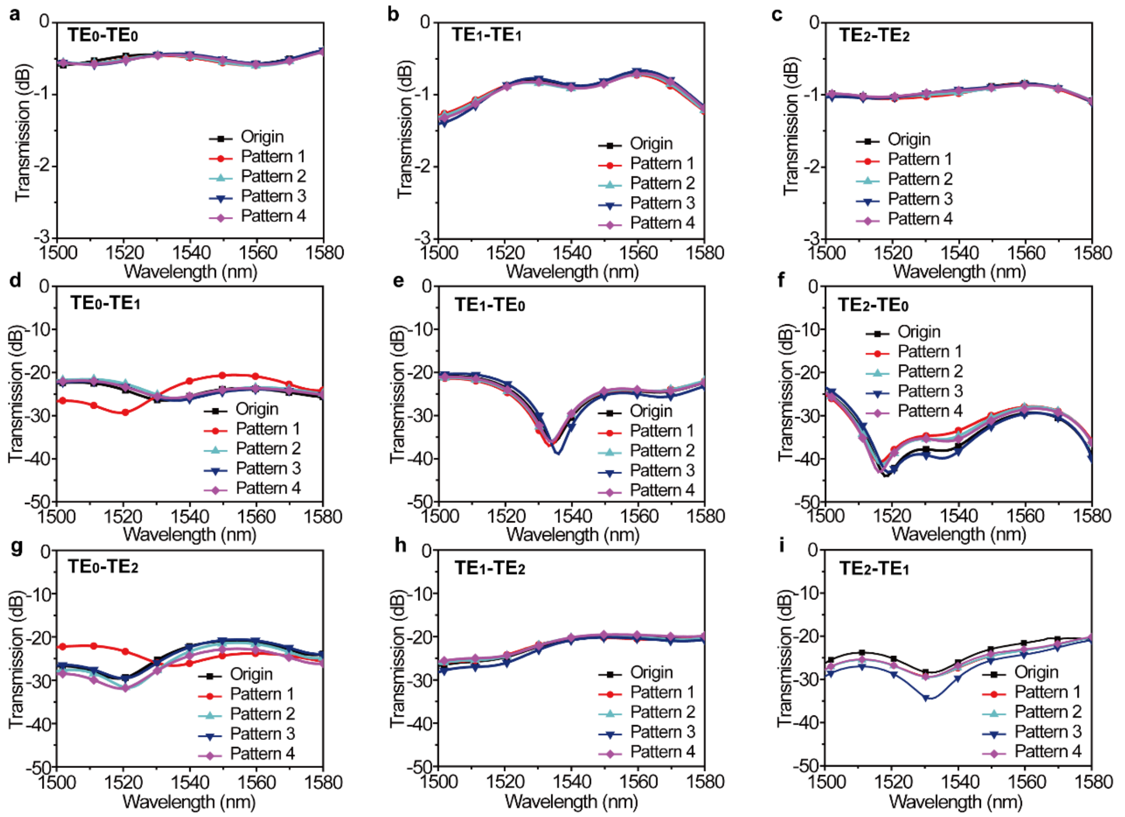

Supplementary Figure 7 | The simulated transmission spectra of the bending with a randomly generated nanohole position errors within 7 nm. (**a**) - (**c**) The simulated ILs for (**a**) TE_0 -TE_0 (**b**) TE_1 –TE_1 (**c**) TE_2 –TE_2. (**d**) - (**i**) The simulated CTs for three different modes.

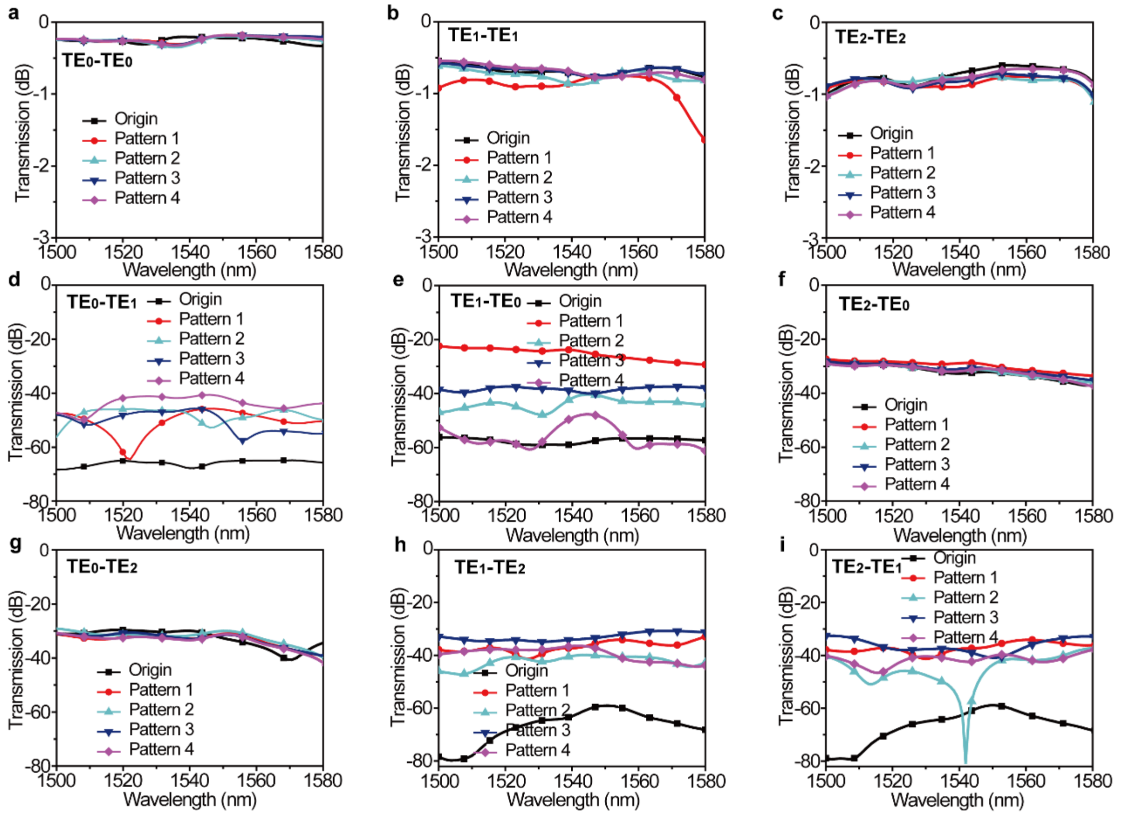

Supplementary Figure 8 | The simulated transmission spectra of the 3-mode crossing with a randomly generated nanohole position errors within 7 nm. (**a**) - (**c**) The simulated ILs for (**a**) TE_0 -TE_0 (**b**) TE_1 –TE_1 (**c**) TE_2 –TE_2. (**d**) - (**i**) The simulated CTs for three different modes.

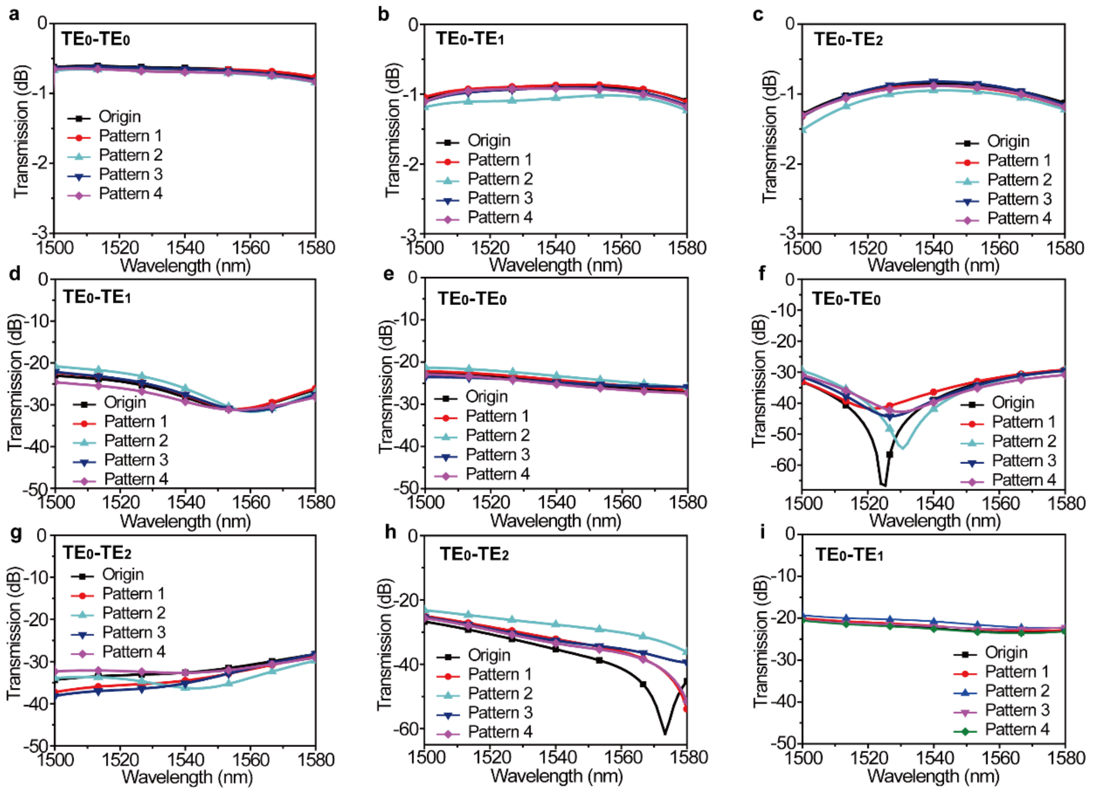

Supplementary Figure 9 | The simulated transmission spectra of the mode (de)multiplexer with a randomly generated nanohole position errors within 7 nm. (**a**) - (**c**) The simulated mode-convert ILs for (**a**) TE_0 -TE_0 (**b**) TE_0 –TE_1 (**c**) TE_0 –TE_2. (**d**) - (**i**) The simulated CTs for three different modes.

3. The fabrication tolerance of the hole shape

The shape of nanohole can be distorted elliptically due to the write field misalignment and deterioration of focus. However, this effect can be well negligible if the E-beam is well optimized. In fact, the nanohole shape will be more easily affected by the sidewall roughness induced by the fabrication imperfection. The a randomly generated error is implemented to the perimeter of each nanohole to simulate the sidewall roughness. We consider the error range from -10 nm to 10 nm. Supplementary Figure 10 describes how we simulate the sidewall roughness. To study the impact of the roughness on the device performance, we randomly generated four patterns with different roughness distributions (defined as Pattern 1, 2, 3, 4). For the waveguide bending, the simulated ILs for nanoholes with different roughness distributions are shown in Supplementary Figure 11 (a), (b), (c) for TE_0, TE_1 and TE_2 mode, respectively. It can be seen that the roughness has negligible impact on the transmission efficiency. The CT performances are also simulated and shown in Supplementary Figure 11 (d)-(i). We found that the device can well tolerate the fabrication error of the nanohole shapes. We have performed similar analysis for the waveguide crossing and (de)MUX, as shown in Supplementary Figure 12 and 13, respectively. The results show that the crossing and MUX devices are quite tolerant to the nanohole shape error as well.

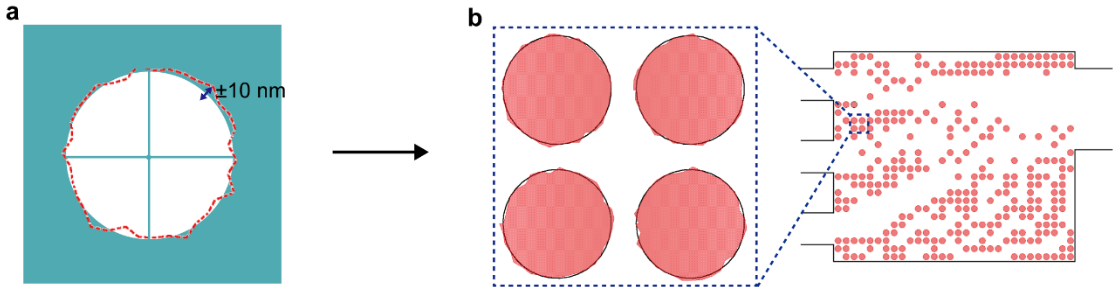

Supplementary Figure 10 | The Schematic illustration of the error in shape of the nanohole. (**a**) Each nanohole has a random roughness within ±10 nm. (**b**) The schematic diagram of the device with randomly generated roughness.

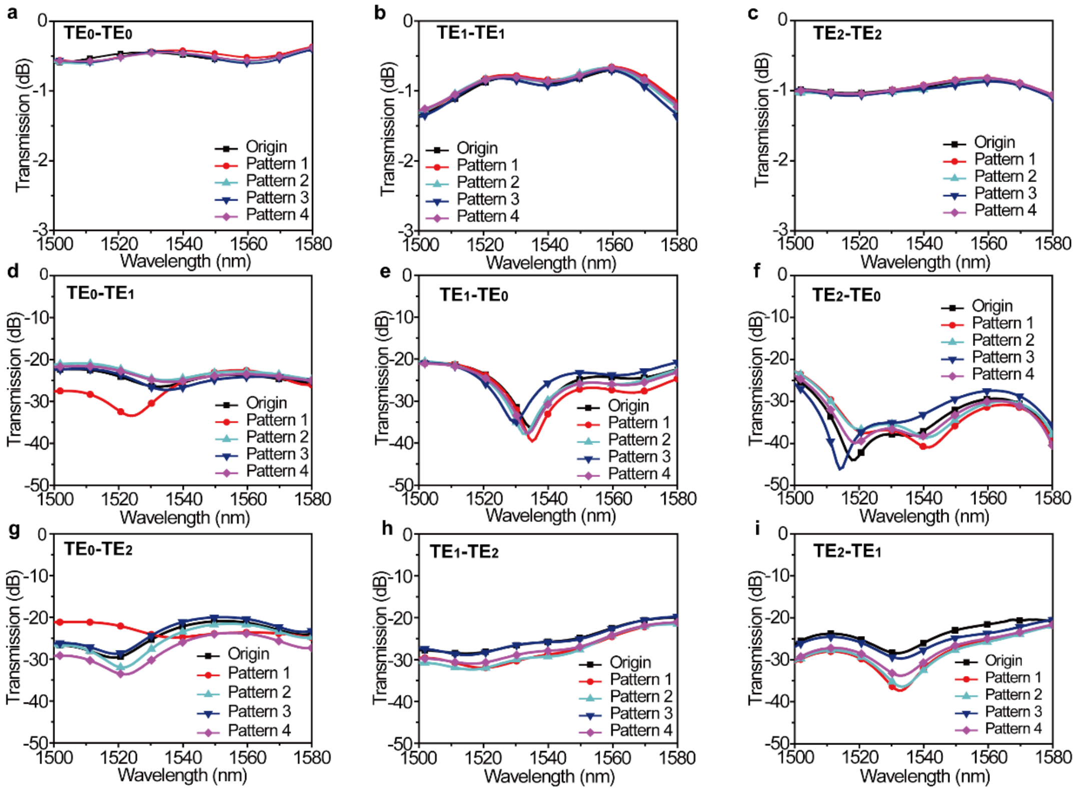

Supplementary Figure 11 | The simulated transmission spectra of the bending with randomly generated roughness. (**a**) - (**c**) The simulated ILs for (**a**) TE_0 -TE_0 (**b**) TE_1 –TE_1 (**c**) TE_2 –TE_2. (**d**) - (**i**) The simulated CTs for three different modes.

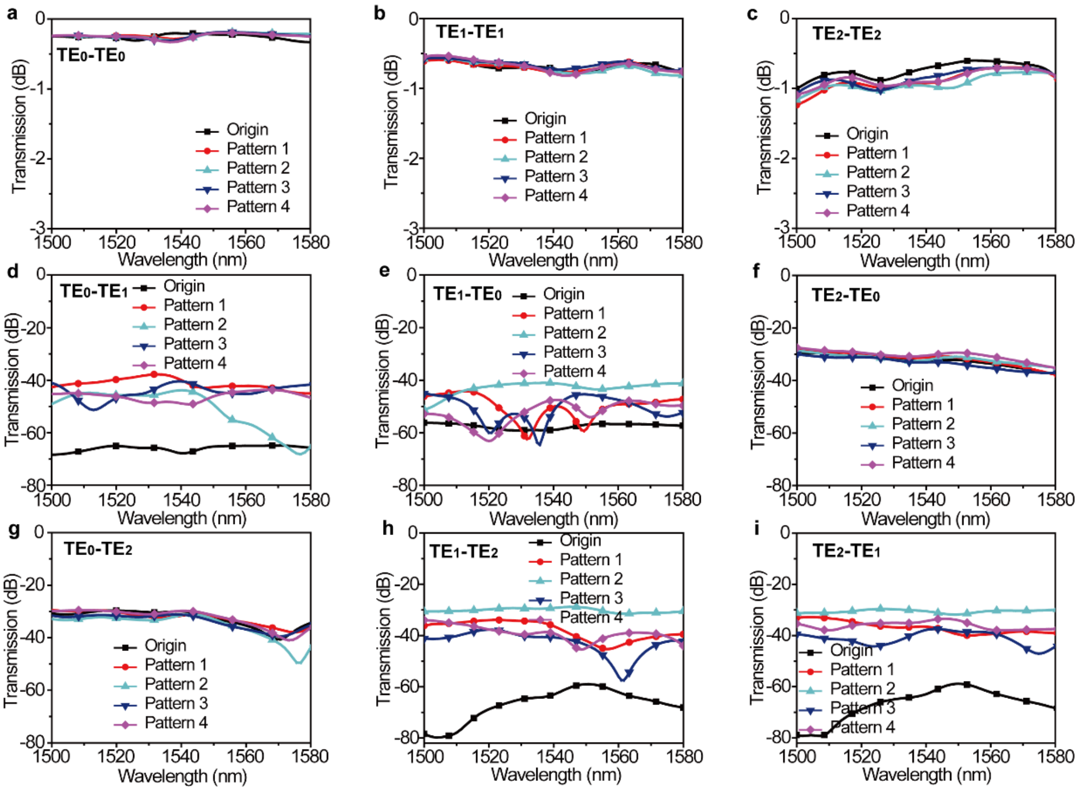

Supplementary Figure 12 | The simulated transmission spectra of the 3-mode crossing with randomly generated roughness. (**a**) - (**c**) The simulated ILs for (**a**) TE_0 -TE_0 (**b**) TE_1 –TE_1 (**c**) TE_2 –TE_2. (**d**) - (**i**) The simulated CTs for three different modes.

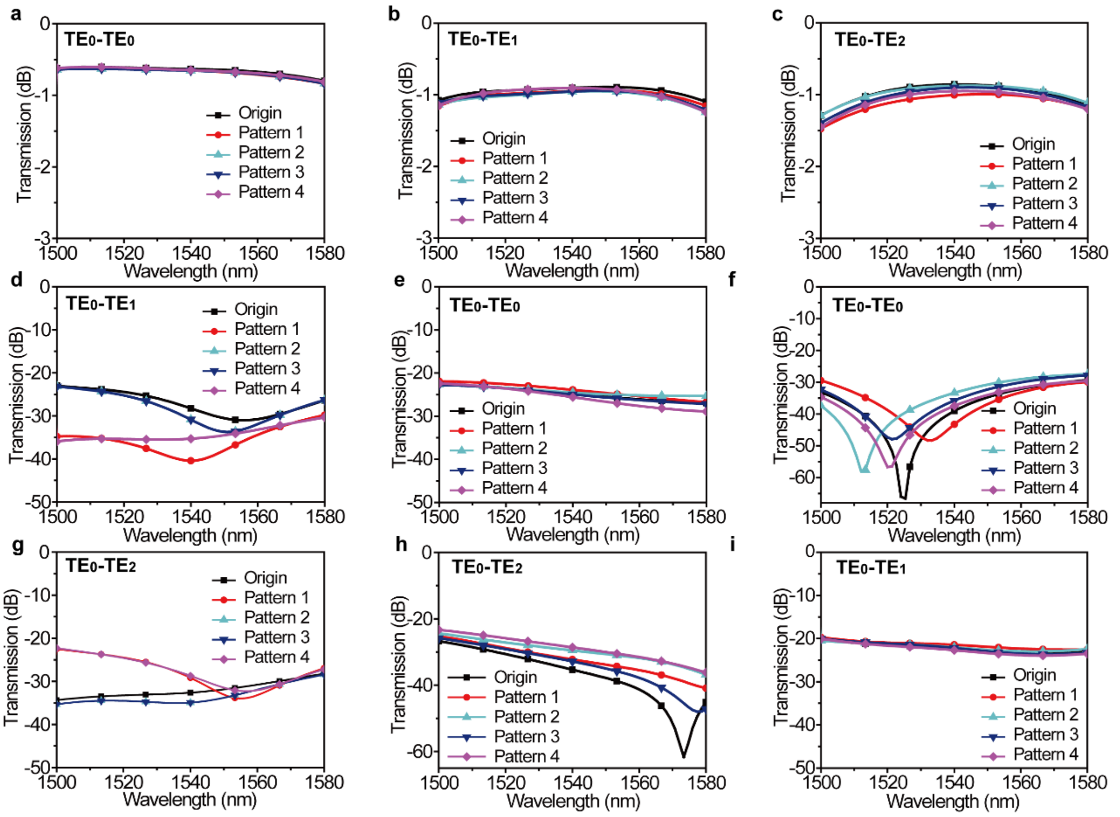

Supplementary Figure 13 | The simulated transmission spectra of the mode (de)multiplexer with randomly generated roughness. (**a**) - (**c**) The simulated mode-convert ILs for (**a**) TE_0 -TE_0 (**b**) TE_0 –TE_1 (**c**) TE_0 –TE_2. (**d**) - (**i**) The simulated CTs for three different modes.

Supplementary Note 4 | 112 Gbit/s signal routing experiment

The experimental setup for the 112 Gbit/s discrete multi-tone (DMT) signal routing is shown in Supplementary Figure 14. The output wavelength of the narrow line-width tunable laser is set to be 1535 nm to match the peak wavelength of the grating coupler. The light is external-modulated by a Lithium Niobate Mach-Zehnder modulator (MZM). The DMT signal is generated by the arbitrary waveform generator (AWG: Keysight, 8195A) with a sample rate of 64 GSa/s, and amplified by an electrical amplifier (EA) to drive the MZM. The optical DMT signal is coupled into the on-chip waveguide through a grating coupler with a single-ended coupling loss of about 6 dB. Erbium doped fiber amplifiers (EDFA) are used before and after the chip for compensating the chip loss, and an optical bandpass filter (OBF) is employed to reduce the Amplified Spontaneous Emission noise after two-state EDFA. The spectrum after OBF is shown in the inset of Supplementary Figure 14. After the MDM transmission, the optical signal is coupled into a single mode fiber and received by a photodetector (PD). The received electrical signal is then sent into a real-time oscilloscope (DSO: Keysight, Z592A) with a sample rate of 160 GSa/s for the off-line digital signal processing (DSP). The DMT signal has a data rate of 112 Gbit/s, with 160 sub-carriers within a bandwidth of 32 GHz. Bit allocation is performed based on Fischer-algorithm according to the signal-noise ratio (SNR) response of the system. The optimized bit allocation is fixed for the back-to-back (B2B) case so that the same DMT signal will be transmitted through the MDM circuits. Thus, the devices can be characterized through the high-speed transmission of the DMT signal.

The raw bitrate of 112 Gbit/s is calculated by 517bits/330*64GSa/s=112Gbit/s, where 517 is the total bit number of one DMT symbol, 330 represents the point number of one DMT symbol which

contains 160 sub-carriers and 10 cyclic prefix, and one DMT symbol maintains 330/64 ns. The calculated bit error rates (BER) under different received power for the DMT signals are shown in Figure 7 (c) in the manuscript, with BER curves well below 20% FEC limit. Given 20% overhead FEC for error-free threshold, the net bitrate is then 93.34 Gbit/s.

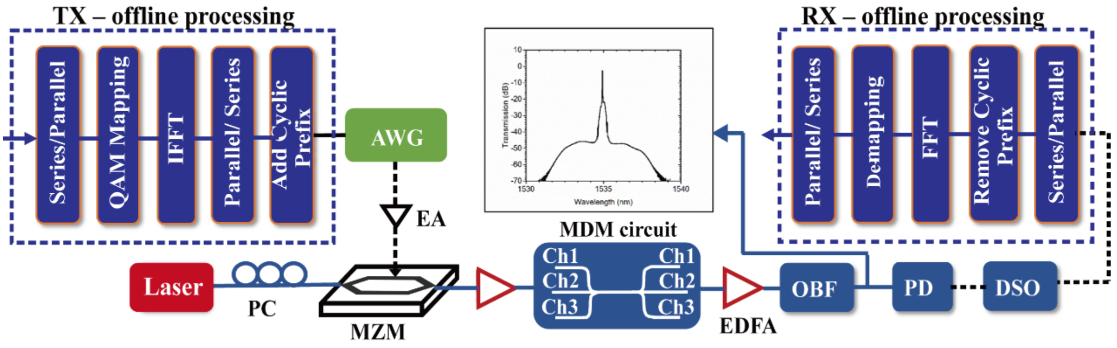

Supplementary Figure 14 | The experimental setup for the on-chip routing of the high-speed signals.

The characterization has also been performed at lower speed of 100 Gbit/s DMT and 80 Gbit/s 4-level pulse-amplitude-modulation (PAM-4). The constellations of sub-carrier (QAM16/32) for 100 Gbit/s and 112 Gbit/s DMT signal under back-to-back, MDM circuit1 transmission, and MDM circuit2 transmission scenarios are shown in Supplementary Figure 15. The constellations of the The slightly degradation of the constellation is due to the decreased SNR induced by the chip insertion loss and inter-modal crosstalk. The results well agree with the BER as shown in Figure 7 (c) in the manuscript.

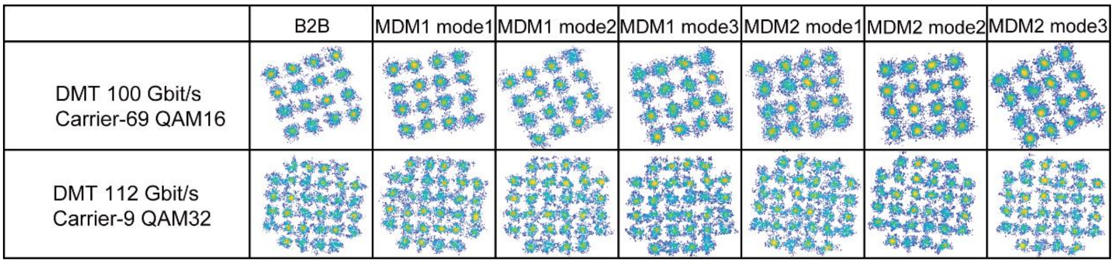

Supplementary Figure 15 | Constellation diagrams of the QAM16 and QAM32 sub-carrier.

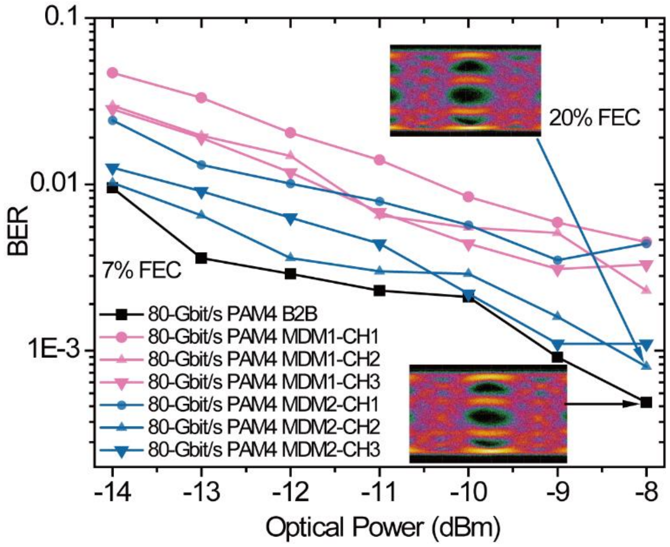

Supplementary Figure 16 | Measured BER curves for B2B and MDM transmission of 80 Gbit/s PAM-4.

The identical pre-distorted PAM-4 signal (without frequency domain bit allocation) at 80-Gbit/s is transmitted in the MDM circuits in order to characterize the devices' performance fairly. The BER curves under different received optical power are plotted in Supplementary Figure 16, and eye diagrams of B2B and MDM circuit2 transmission scenarios are depicted in the insets. The BERs are well below the 20% FEC limit. We can see from the eye diagrams that MDM transmission does not bring in obvious degerenarion.

Supplementary Note 5 | Compatibility with silicon photonic foundry fabrication

Here, we demonstrate the devices' compatibility with silicon photonics foundry fabrication. We follow IMEC's design rule (hole size: 130 nm in diameter; nanohole gap distance: 120 nm; oxide cladding) and re-optimize the devices. Supplementary Figure 17 (a) illustrates the schematic diagram and the structural parameters of the re-designed bending with oxide cladding. The simulated optical field distribution of the bending for TE_0, TE_1 and TE_2 at 1550 nm are shown in Supplementary Figure (b) – (d), respectively. Supplementary Figure 17 (e) - (g) show the simulated transmission spectra of the bending structure for TE_0 - TE_2 from 1500 to 1580 nm. For TE_0 - TE_2, the ILs of all modes are less than 1 dB and the CTs are lower than -20 dB. Similarly, we re-design the waveguide crossing and (de)MUX following IMEC's design rule. The device schematic diagram, optical field distributions, transmission curves of the crossing and (de)MUX are shown in Supplementary Figure 18 and 19, respectively. For all the modes, the ILs are less than 1 dB and the CTs are lower than -20 dB for both devices. Hence, the MDM devices proposed in this work can be fully compatible with the design rule given by the silicon photonics foundry.

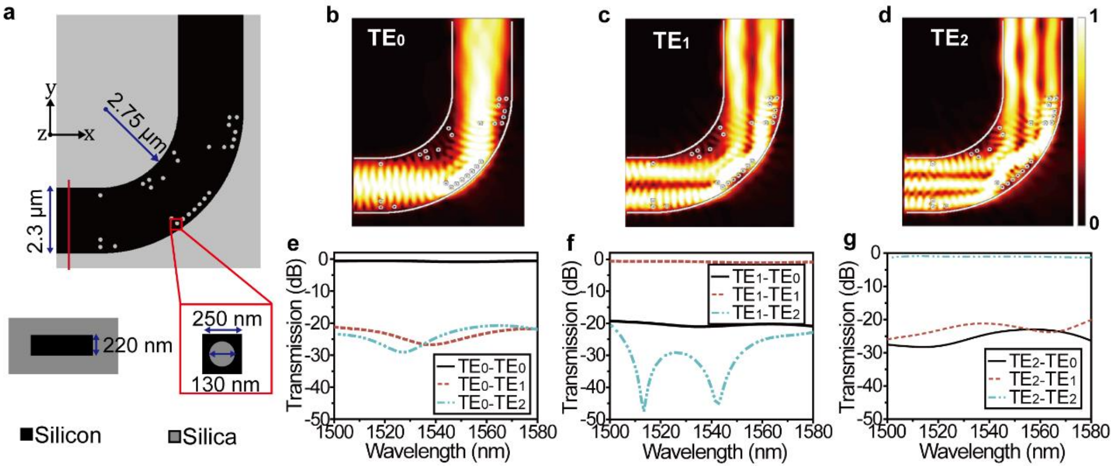

Supplementary Figure 17 | Design and simulation results of the bending following the foundry's design rule. (a) Schematic of the optimized bending following IMEC's design rule. (b) - (d) The simulated optical field distribution of the bending for TE_0 - TE_2 at 1550 nm. (e) - (g) The simulated transmission spectra of the bending for TE_0 - TE_2 from 1500 to 1580 nm.

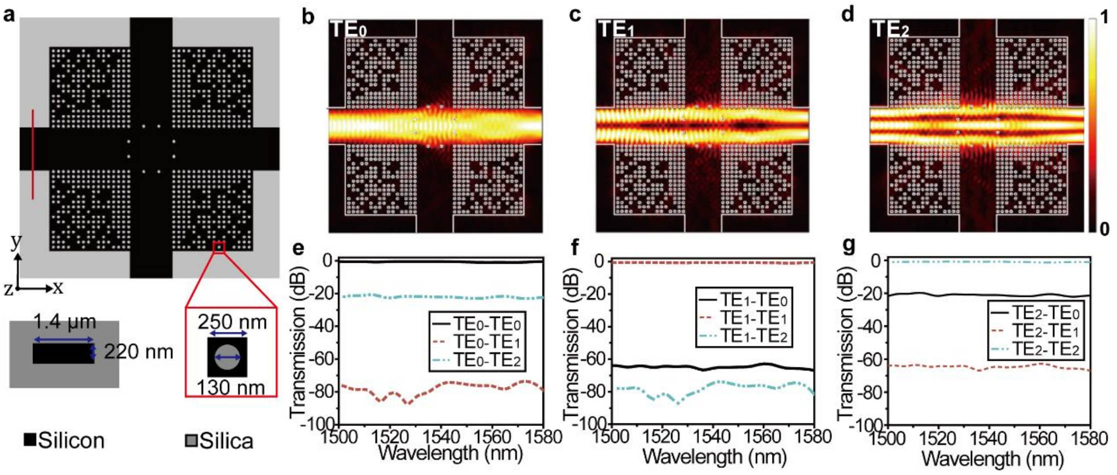

Supplementary Figure 18 | Design and simulation results of the crossing following the foundry's design rule. (a) Schematic of the optimized crossing following IMEC's design rule. (**b**) - (**d**) The simulated optical field distribution of the crossing for TE_0 - TE_2 at 1550 nm. (**e**) - (**g**) The simulated transmission spectra of the crossing for TE_0 - TE_2 from 1500 to 1580 nm.

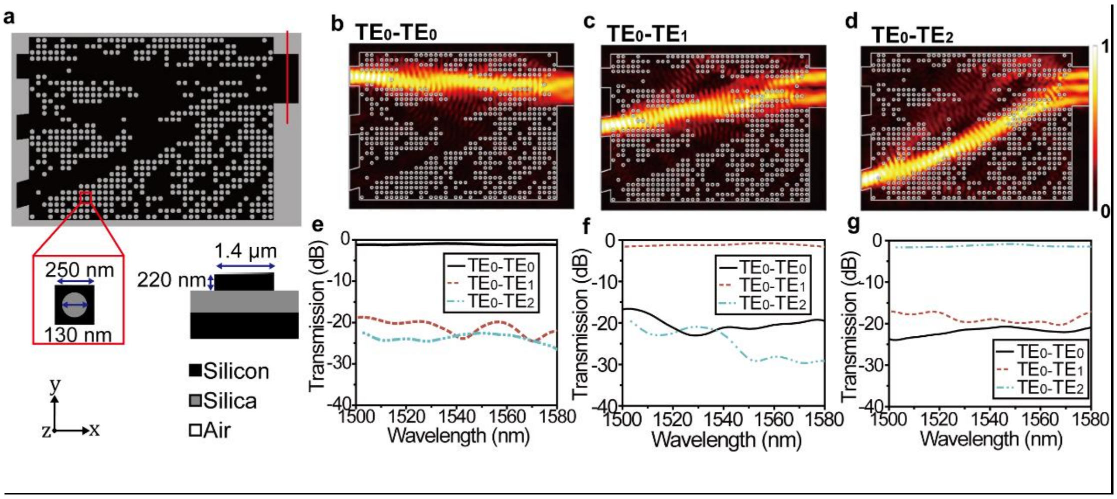

Supplementary Figure 19 | Design and simulation results of the MUX following the foundry's design rule. (a) Schematic of the optimized MUX following IMEC's design rule. (**b**) - (**d**) The simulated optical field distribution of the (de)MUX for TE_0 - TE_2 at 1550 nm. (**e**) - (**g**) The simulated transmission spectra of the (de)MUX for TE_0 - TE_2 from 1500 to 1580 nm.

Supplementary Note 6 | The contributions from nanoholes

For the inversely designed digital meta-devices, the functionalities are realized by an overall contribution from all the nanoholes. To confirm this point, we simulated the device performance for the bending and crossing with the same geometry, but all the holes are filled by silicon. Supplementary Figure 20 (a) - (b) are the schematic diagrams of the 3-mdoe waveguide bend without and with nanoholes, respectively. The Supplementary Figure 20 (c) is the simulated transmission spectral result of proposed micro-bend without nanoholes for TE_0-TE_2 from 1500 to 1580 nm. The transmission efficiencies drop to about 5dB for all modes. This degradation is confirmed by the optical field distributions as shown in Supplementary Figure 20 (d) - (f). Significant inter-mode coupling occurs at the bending region as well.

Supplementary Figure 21 (a) - (b) are the schematic diagram of the proposed crossing without and with nanoholes, respectively. The transmission spectra of crossing without nanoholes for TE_0-TE_2 from 1500 to 1580 nm are shown in Supplementary Figure 21 (c). The ILs increase significantly for higher

order modes due to the inter-mode coupling. This is confirmed by the simulated optical field distribution shown in Supplementary Figure 21 (d) - (f). Furthermore, we simulated the light field distribution when two optical waves were launched from two orthogonal input waveguides as shown in Supplementary Figure 22 (a) and (e). A comparison is also made between the DMS and the crossing without nanoholes, as shown in Supplementary Figure 22 (b) – (d) and (f) – (h).

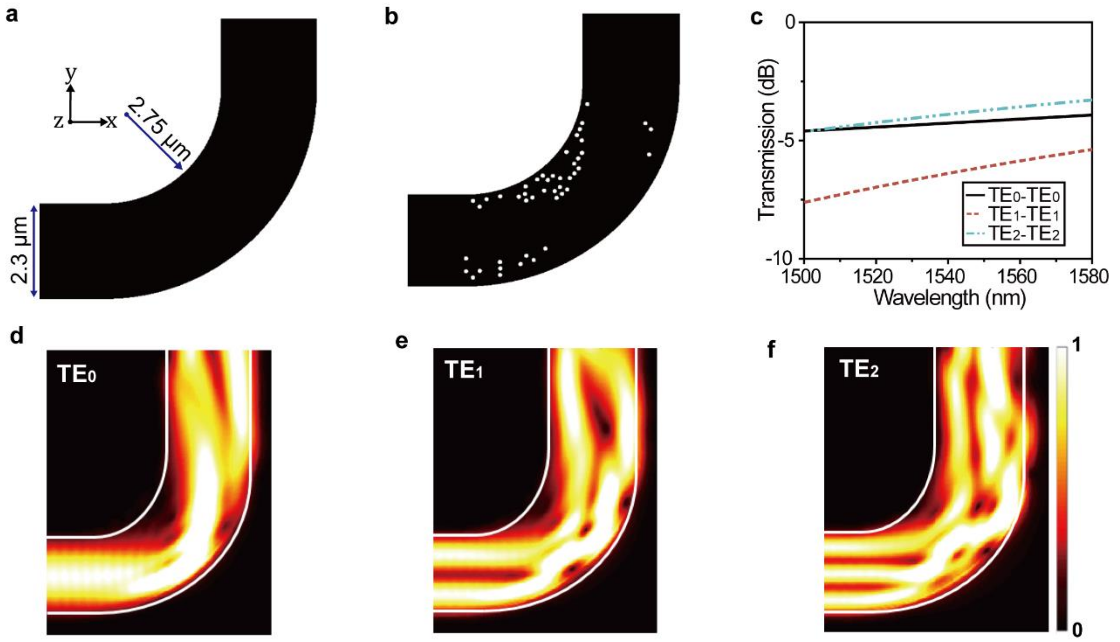

Supplementary Figure 20 | Simulation results of the bending without nanoholes. (**a**) The schematic of the bending (**a**) without nanoholes, (**b**) with nanoholes. (**c**) The simulated transmission spectra of the bending without nanoholes for TE_0 - TE_2 from 1500 to 1580 nm. (**d**) - (**f**) The simulated optical field distribution of the bending without nanoholes for TE_0 - TE_2 at 1550 nm.

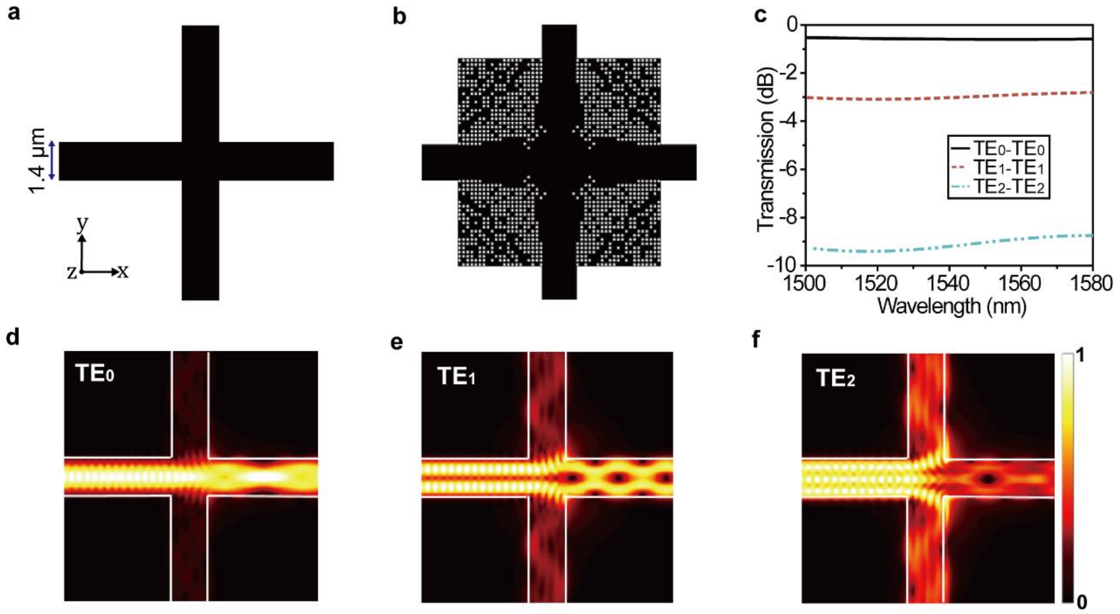

Supplementary Figure 21 | The simulation results for the crossing without nanoholes. The schematic of the crossing (**a**) without nanoholes, (**b**) with nanoholes. (**c**) The simulated transmission spectra of the crossing without nanoholes for TE_0 - TE_2 from 1500 to 1580 nm. (**d**) - (**f**) The simulated optical field distribution of the crossing without nanoholes for TE_0 - TE_2 at 1550 nm.

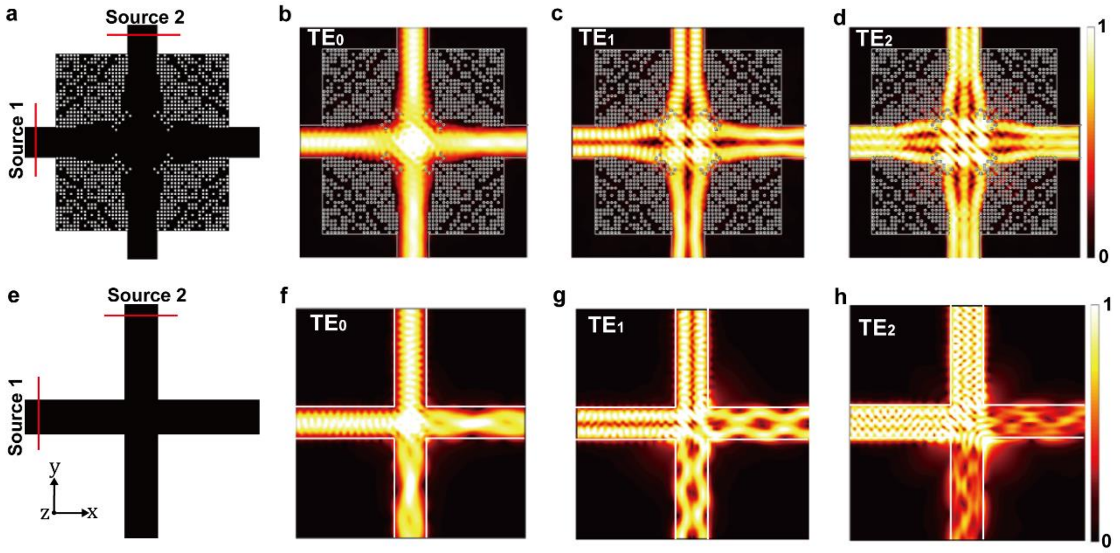

Supplementary Figure 22 | Performance comparison between the crossing with and without nanoholes when two waves are launched in orthogonal directions. The schematic diagram of the device and the simulated optical field distributions of the (**a**) crossing with nanoholes, (**b**) crossing without nanoholes.
